# QseC Inhibition as a Novel Antivirulence Strategy for the Prevention of Acute Hepatopancreatic Necrosis Disease (AHPND)-Causing *Vibrio parahaemolyticus*

**DOI:** 10.3389/fcimb.2020.594652

**Published:** 2021-01-21

**Authors:** Qian Yang, Peizhuo Zou, Zhi Cao, Qingyao Wang, Songzhe Fu, Guosi Xie, Jie Huang

**Affiliations:** ^1^ Laboratory for Marine Fisheries Science and Food Production Processes, Qingdao National Laboratory for Marine Science and Technology, Qingdao, China; ^2^ Key Laboratory of Maricultural Organism Disease Control, Ministry of Agriculture and Rural Affairs, Qingdao, China; ^3^ Qingdao Key Laboratory of Mariculture Epidemiology and Biosecurity, Yellow Sea Fisheries Research Institute, Chinese Academy of Fishery Sciences, Qingdao, China; ^4^ Center for Microbial Ecology and Technology (CMET), Ghent University, Gent, Belgium; ^5^ College of Marine Science and Environment, Dalian Ocean University, Dalian, China; ^6^ Key Laboratory of Environment Controlled Aquaculture (KLECA), Ministry of Education, Dalian, China; ^7^ Network of Aquaculture Centers in Asia-Pacific, Bangkok, Thailand

**Keywords:** acute hepatopancreatic necrosis disease, *Vibrio parahaemolyticus*, antivirulence therapy, QseC, LED209

## Abstract

Acute hepatopancreatic necrosis disease (AHPND) caused by *Vibrio parahaemolyticus* resulted in great economic losses in global shrimp aquaculture. There is an urgent need for development of novel strategies to combat AHPND-causing *V. parahaemolyticus* (*Vp*_AHPND_), given that one of the greatest challenges currently is the widespread use of antibiotics and subsequent emergence of multidrug-resistant bacteria. Here, we proposed a broad-spectrum antivirulence approach targeting a conserved histidine kinase, QseC, which has been demonstrated to activate virulence expression in several Gram-negative pathogens. Our results showed that QseC mediated the catecholamine stimulated effects on growth and flagellar motility of *Vp*_AHPND_. Transcriptome analysis revealed that QseC was involved in the global regulation of the virulence of *Vp*_AHPND_ as the Δ*qseC* mutant exhibited a decreased expression of genes related to type IV pilin, flagellar motility, and biofilm formation, while an overexpression of type VI secretion system and cell wall biosynthesis. Subsequently, the bacterial catecholamine receptor antagonist LED209 not only neutralized the stimulatory effects of host catecholamines on the growth and motility of *Vp*_AHPND_ *in vitro*, but also attenuated the virulence of *Vp*_AHPND_ towards brine shrimp larvae and white shrimp *in vivo*. Additionally, LED209 presented no interference with pathogen growth, nor the toxicity to the experimental animals. These results suggest that QseC can be an attractive antivirulence therapy target, and LED209 is a promising candidate for development of broad-spectrum antivirulence agents. This is the first study that demonstrated the role of QseC in the global regulation of *Vp*_AHPND_ infection and demonstrated the antivirulence potential of LED209, which provides insight into the use of an antivirulence approach for targeting not only *Vp*_AHPND_, but also a much larger collection of pathogenic bacteria.

## Introduction

*Vibrio parahaemolyticus* is a gram-negative, halophilic bacterium that is disseminated worldwide in marine and estuarine environments (Jones, 2014). It is frequently isolated from environmental, seafood and clinical samples, and is considered to be the leading cause of human foodborne illness in the United States and Asian countries (Newton et al., 2012; Yu et al., 2013). Specific virulent strains of *V. parahaemolyticus*, which contain a ~70 kb plasmid with genes encoding homologues of the Photorhabdus insect-related (Pir) binary toxin PirAB^VP^, have recently been identified as the causative agent of acute hepatopancreatic necrosis disease (AHPND) in global shrimp industry (Tran et al., 2013). AHPND can lead to mass mortalities (up to 100%) within the first 30–35 days after stocking shrimp post-larvae or juveniles in grow-out ponds (FAO, 2013). Till now, multiple shrimp species have been listed as being susceptible to AHPND-causing vibrios, including giant tiger prawn (*Penaeus monodon*), whiteleg shrimp (*Penaeus vannamei*), and fleshy prawn (*Penaeus chinensis*) (Joshi et al., 2014; Hong et al., 2016; Reantaso, 2016).

Research in terms of AHPND has been mostly focused on understanding its pathogenesis and identifying potential pathogens. Due to the current shortage of effective drugs in the shrimp farming practices, the widespread use of antibiotics and subsequently emergence of multidrug-resistant bacteria are increasing at an alarming rate and constitute one of our greatest challenges in combating AHPND-causing *V. parahaemolyticus* (*Vp*_AHPND_). Therefore, the development of alternative antimicrobial therapies to remedy this disease is urgently needed.

Among the novel therapeutic strategies, antivirulence therapy has emerged as a promising alternative. Instead of directly killing the bacteria, this strategy is aimed at depriving them from their essential virulence factors and preventing the attack on the host. Thus, it would exert a relatively mild selective pressure over pathogens than that exerted by the classical bactericidal or bacteriostatic antibiotic activities (Defoirdt, 2013; Vale et al., 2016; Dickey et al., 2017; Munguia and Nizet, 2017). In addition, the spread of resistance determinants through horizontal gene transfer would also be reduced since most virulence factors are usually restricted to a single few closely related species. Moreover, another encouraging potential of antivirulence agents is that they have less impact on the normal microbiota of the host, overcoming undesirable side effects of traditional antibiotic therapy (Buroni and Chiarelli, 2020).

A variety of virulence factors have been currently under investigation as potential antivirulence targets, such as adhesion and motility, biofilm formation, siderophores, and secretion systems (Donlan and Costerton, 2002; Gerlach and Hensel, 2007; Skaar, 2010; Maier and Wong, 2015). Since the production of virulence factors is energetically costly, the expression of virulence genes is usually controlled by complex regulatory mechanisms; therefore, disruption of these regulatory mechanisms could affect the production of several virulence factors (Defoirdt, 2018). Furthermore, the signal receptor proteins of bacteria are usually highly conserved, indicating that the same signal inhibitor can inhibit multiple bacteria and strains, reflecting the broad-spectrum nature of this method (Defoirdt, 2014). Given these properties, the possibility to interfere with the regulatory networks of multiple virulence determinants would be one of the most promising targets for antivirulence therapies. Among them, signaling pathways such as quorum sensing and two-component sensing have gained particular attention (Defoirdt, 2018).

A broad spectrum of bacterial pathogens rely on QseC, which is a conserved membrane-bound histidine sensor kinase belonging to the QseBC two-component system, to recognize the host environment by sensing and responding to the host catecholamine stress hormones in order to promote the expression of different virulence factors (Rasko et al., 2008; Weigel and Demuth, 2016). The catecholamine stress hormones epinephrine (EPI), norepinephrine (NE), and dopamine (DA) are an integral part of the stress response in humans (Reiche et al., 2004) and stimulate the growth of several pathogens in serum-based media (Lyte and Ernst, 1992; Coulanges et al., 1997; Yang et al., 2014; Boyanova, 2017). Suong et al. (2017) also demonstrated that the catecholamines NE and DA could significantly increase the motility and virulence of *Vp*_AHPND_.

Catecholamines exert their effects by binding to specific receptors. Several bacterial catecholamine receptors have also been reported, including the histidine sensor kinases QseC (Rasko et al., 2008). QseC homologs are widely present in at least 25 important human and plant pathogens, such as enterohemorrhagic *Escherichia coli* (EHEC; O157:H7), *Salmonella enterica*, uropathogenic *E. coli*, *Haemophilus influenza*, *Aeromonas hydrophila*, *Edwardsiella tarda*, and *Francisella tularensis* (Weiss et al., 2007; Bearson and Bearson, 2008; Kostakioti et al., 2009; Wang et al., 2011; Khajanchi et al., 2012; He et al., 2018). Thus, QseC represents a novel target for antivirulence strategy, and small molecule inhibitors have already been reported to show promise as broad-spectrum antimicrobials.

LED209 [*N-*phenyl-4-(3-phenylthioureido)benzenesulfonamide] is the first reported bacterial catecholamine receptor antagonist (Rasko et al., 2008). It blocks the autophosphorylation of QseC and thus blocks its binding to catecholamines, thereby inhibiting activation of the expression of several virulence genes mediated by QseC. LED209 has exhibited good potential for antivirulence activity of several bacterial pathogens, including *S. enterica* ser. Typhimurium, *F. turalensis*, adherent-invasive *E. coli*, and *V. harveyi* (Rasko et al., 2008; Curtis et al., 2014; Yang et al., 2014).

Catecholamine sensing has been reported to affect the virulence of a *Vp*_AHPND_ strain isolated from outbreaks in Vietnam and LED209 has been proved to be effective for the inhibition of this moderate-virulence strain (LD_50_ = 6 × 10^6^ CFU/ml^-1^) (Suong et al., 2017). In this article, we examined i) whether QseC mediates catecholamine-induced effects in *Vp*_AHPND_; and ii) whether QseC blockade with LED209 could be a promising antivirulence therapy for a high virulent *Vp*_AHPND_ strain (LD_50_ = 5 × 10^4^ CFU/ml^-1^). This work may shed lights on a better understanding of factors involved in AHPND epidemiology and provide insight into the development of potential antivirulence agents for the prevention and control of AHPND.

## Materials and Methods

### Bacterial Strains and Growth Conditions

A *Vp*_AHPND_ strain 123 was used in this study, which previously isolated from an AHPND outbreak and caused 100% shrimp mortality in Hebei province of China. Bacterial strains and plasmids used in this study were listed in **Table 1**. Unless otherwise stated, *Vp*_AHPND_ strains were cultured at 28°C in Tryptic Soy Broth containing 30 g/L^−1^ NaCl (TSB30) under constant agitation (100 min^−1^), while *Escherichia coli* strains were cultured in Luria broth (LB; Difco) at 37°C. Cell densities were measured spectrophotometrically at 600nm.When required, ampicillin (Amp), chloramphenicol (Cm), IPTG, X-Gal and L-arabinose were supplemented at final concentrations of 100, 34, 100, 50 µg ml^−1^ and 2 mg ml^−1^, respectively.

**Table 1:** Strains and plasmids used in this study.

Strains	Description	Source
*Vibrio parahaemolyticus*		
*Vp*_AHPND_ 123	Wild type, isolated from *Penaeus vannamei*, China, Cm^s^, Amp^r^	This study
Δ*qseC*	*Vp*_AHPND_ 123, in-flame deletion of *qseC*	This study
*qseC+*	*Vp*_AHPND_ 123, Δ*qseC*, containing pC*qseC*	This study
		
*Escherichia coli*		
SY327 (λpir)	Host strain for π-dependent plasmids	Miller and Mekalanos, 1988
		
*Plasmids*		
pRE112	Suicide vector, π-dependent, *sacB*, Cm^r^	Edwards et al., 1998
pUCm-T	Cloning vector, Amp^r^	Sangon, Shanghai
pdCas9-bacteria	Expressing vector, Cm^r^	Addgene 44249
pRE112Δ*qseC*	pRE112 derivative for *qseC* in-frame deletion	This study
pC*qseC*	QseC expressing plasmid, Cm^r^	This study

### Catecholamines and Bacterial Catecholamine Receptor Antagonist

Epinephrine hydrochloride (EPI) and Dopamine hydrochloride (DA) were dissolved in distilled water at 10 mM and were sterilized using a 0.22 μM filter, while norepinephrine (NE) and LED209 were dissolved in DMSO at 10 mM. EPI and DA were purchased from Sigma-Aldrich, Missouri, USA. NE and LED209 were obtained from Cayman Chemical, Michigan, USA. The reagents were stored at -20°C.

### Construction of *qseC* In-Frame Deletion Mutant *Vp*_AHPND_ 123Δ*qseC*

A *qseC* in-frame deletion (Δ*qseC*) mutant of *Vp*_AHPND_ strain 123 was constructed by double crossover allelic exchange using suicide vector pRE112. In brief, two pairs of primers, *qseC*-LF/*qseC*-LR and *qseC*-RF/*qseC*-RR (**Table 2**), were applied to amplify fragments upstream (797 bp) and downstream (668 bp) of *qseC* gene from chromosomal DNA, respectively. Both fragments were purified and fused in a subsequent overlap PCR using primers *qseC*-LF and *qseC*-RR. The fused segment (Δ*qseC*) was ligated into pRE112 at HindIII site and introduced into *E. coli* SY327 (λpir). The resulting plasmid, pRE112Δ*qseC*, was verified by PCR and sequencing and then introduced into *Vp*_AHPND_ 123 by electroporation. The positive clone with pRE112Δ*qseC* integrated into the chromosome by a single crossover event was selected on TSA30 (Tryptic Soy Agar with 30g L^-1^ NaCl) containing ampicillin and chloramphenicol with primers Check-F/R. Then the excision of pRE112 from the chromosome was achieved by a second crossover with counter-selection on TSA30 containing 10% sucrose. The resulting Δ*qseC* mutant was verified by PCR and sequencing.

**Table 2:** Primers used in this study.

Primers	Sequences (5’-3’)	References
**Construction of mutant and complementary strain**		
*qseC*-LF	TCG**AAGCTT**AGAAGCGAAAGTGTATTTGAC	This study
*qseC*-LR	CTATTGTTGCGCGTTTAATGGAGTAAGGTTT	
*qseC*-RF	CATTAAACGCGCAACAATAGAGCTGATTCC	This study
*qseC*-RR	CAT**AAGCTT**TTTCGGGTAGATGAGGTATTC	
Check-F	GCAGAAGAAGTCATTCAAGTT	This study
Check-R	GGATGAACAACCAGCATAAC	
C-F	CAT**CTTAAG**GAACTATTTTTATGGAGACTTCCTTG	This study
C-R	CTT**CTCGAG**CTAGTACTTTGGGAATGCGAC	
		
**Quantitative real-time reverse transcription PCR**		
*recA*-F	GCTAGTAGAAAAAGCGGGTG	Ma et al., 2015
*recA*-R	GCAGGTGCTTCTGGTTGAG	
*qseC*-F	TCAGCCAGAACAGCAACA	This study
*qseC*-R	AGACCGAGCATGAGAACG	
*flaC*-F	ACCGTATCGCAGAAACCACA	This study
*flaC*-R	GCCAGGAAGCGTGATTTTTA	
*flaF*-F	TTTGGATGTGGCGGTTCG	This study
*flaF*-R	GCTCGGCTTGGCTGTTTG	
*flaK*-F	GCGTATGGTCAGGCTGTATC	This study
*flaK*-R	TGTGGGTAATTTAGCGGTTG	
*fliA*-F	CCGACGAGCAAGATTATTACA	This study
*fliA*-R	ATCCAAGACGAGGGCTATTT	
*motB*-F	CTTTACGGTGCGGTGTTG	This study
*motB*-R	GCGGTTAAGCGTTTCTTG	
*cpsA*-F	GTATTACTCCGTTTGGTGG	This study
*cpsA*-R	CTGGTTTGACTTTGTGGC	
*vasD*-F	ATGCTCCGCCGCTAAT	This study
*vasD*-R	ACAACTGGTGATGGTCTTC	

### Construction of Complementary Strain *Vp*_AHPND_ 123*qseC+*

To construct a complementary strain of *Vp*_AHPND_ 123Δ*qseC*, the intact gene containing the putative promoter region was amplified by primers C-F and C-R and introduced into pdCas9-bacteria (addgene 44,249) at AflII/XhoI sites. The resulting QseC-expression plasmid, designated pC*qseC*, was then introduced into *Vp*_AHPND_ 123Δ*qseC* by electroporation and verified by PCR and sequencing, resulting the complementary strain *Vp*_AHPND_ 123*qseC*+.

### Genome-Wide Transcriptomic Analysis of the Wild Type and Δ*qseC* Mutant

Wild type and Δ*qseC* mutant strains were grown in Dulbecco’s modified Eagle medium (DMEM; Invitrogen Life Technologies, Carlsbad, CA) with shaking at 100 rpm for 24 h. The total RNA was extracted using the RNeasy Protect Bacteria Mini Kit (Qiagen, Hilden, Germany) as per the manufacturer’s recommendation, and paired-end sequencing was performed on the Illumina Hi-Seq 2000 platform of the LC-Bio Technology Co., Ltd. (Hangzhou, Zhejiang, China). The raw transcriptomic sequencing data were submitted to GenBank (NCBI) under the BioProject No. PRJNA668022. Raw reads of transcriptome sequencing were mapped to genome 123 by Bowtie2-2.2.3. Differential expression analysis was performed for three biological replicates of two strains using the DESeq R package (1.18.0). Genes were considered differentially expressed with over 1.5-fold change and *p*-values <0.01 in samples.

### Quantitative Reverse Transcription PCR (RT-qPCR)

To analyze the gene expression of seven genes, including *qseC*, *cpsA* (relating to biofilm formation), *flaF* and *flaC* (encoding polar flagellins), *flaK* (encoding σ^54^-interacting regulator), *fliA* (encoding polar flagellin specific chaperon), *motB* (encoding Na^+^ motor component), and *vasD* (associating with T6SS) between wild type and mutant strains, we performed RT-qPCR using methods described by Fu et al. (2020). The RT-qPCR assay was performed on three technical and three biological replicates for each sample. The expression levels of these genes were normalized with the house keeping gene *recA.* The fold changes relative to *recA* were calculated using the 2^-ΔΔCT^ method (Livak and Schmittgen, 2001). RT-qPCR data of three independent experiments are showed as the Mean ± SD (standard deviation).

### Bacterial Growth Assays

Standard American Petroleum Institute (SAPI) medium with serum was employed in this study to mimic the *in vivo* conditions within a host (Lyte and Ernst, 1992). Serum-SAPI medium contains limited nutrients, including 6.25 mM NH_4_NO_3_, 1.84 mM KH_2_PO_4_, 3.35 mM KCl, 1.01 mM MgSO_4_, and 2.77 mM glucose, supplemented with 30% (v/v) adult bovine serum (Biological industries, Israel). The medium pH was adjusted to 7.5 and filter-sterilized.

Different *Vp*_AHPND_ strains were grown overnight in TSB30 at 28°C, after which the cultures were re-inoculated at a concentration of 10^2^ CFU ml^−1^ into fresh serum-SAPI medium, with and without EPI (100 μM), NE (50 μM), or DA (50 μM). Additionally, different concentrations of bacterial catecholamine receptor antagonist were added in conjunction with the catecholamines to determine whether it could neutralize catecholamine-induced growth responses. The cultures were grown statically in 30 ml volumes at 28°C for 48 h in a 5% CO_2_ humidified incubator, after that the number of bacteria was enumerated by standard pour-plate analysis using TSA30 (Freestone and Lyte, 2008). Controls comprised equivalent volumes of the solvent used to dissolve the catecholamines or the antagonists. Growth response assays were determined for three independent cultures, and all experiments were conducted on at least three separate occasions.

Growth curves of different strains were also determined. Overnight cultures were prepared for all strains (i.e., *Vp*_AHPND_ 123 wild type, Δ*qseC* mutant and complementary strain *qseC+*), then the cultures were re-inoculated into fresh TSB 30. Additionally, catecholamine receptor antagonist LED209 and equivalent volume of the solvent (DMSO) were added to evaluate whether they could affect the growth of *Vp*_AHPND_ 123. The cultures were grown at 28°C for 36 h, and the turbidity at 600 nm was monitored every three hours. Growth curves were determined for three independent cultures.

### Swimming Motility Assay

The swimming motility assay was performed on soft agar (TSA 30 plates containing 0.3% agar) as described previously (Yang and Defoirdt, 2015). The catecholamines and antagonist were directly added to the autoclaved agar. *Vp*_AHPND_ strains were grown in TSB30 until OD_600_ = 1.0, and 2 μl aliquots were spotted in the center of the soft agar plates. The diameters of the motility halos were measured after incubation at 28°C for 18 h. All assays were conducted with freshly prepared plates in three replicates.

### Biofilm Formation Assay

Biofilm formation assay was quantified using crystal violet staining as described by Yang et al. (2014). In brief, overnight cultures of *Vp*_AHPND_ strains were diluted to an OD_600_ of 0.1 in TSB30, and were pipetted into a 96-well plate with 200 μl aliquots of each well. Then the plate was incubated without agitation for 24 h at 28°C to allow the bacteria adhere and grow. After then the cultures were removed and the wells were washed three times with 300 μl sterile physiological saline. The remaining attached bacteria were fixed with 200 μl of methanol per well for at least 20 min, after which the methanol was removed and plates were air-dried. Subsequently, biofilms were stained with 200 μl of a 0.1% crystal violet solution (Sigma-Aldrich, Missouri, USA) per well for 15 min. Excess stain was rinsed off by placing the plate under running tap water, and washing was continued until the washings were free of the stain. After the plates were air dried, the dye bound to the adherent cells was resolubilized with 200 μl of 95% ethanol per well, and absorbance was measured at 570 nm. Sterile medium served as negative control.

### Axenic Hatching of Brine Shrimp Larvae and Brine Shrimp Challenge Test

Five hundred milligrams of high-quality hatching cysts of *Artemia franciscana* (EG^®^ Type; INVE Aquaculture, Baasrode, Belgium) were hydrated in 45 ml of sterile distilled water for 1 h. Sterile cysts were obtained by decapsulation according to the method described by Marques et al. (2005). Briefly, 1.65 ml of NaOH (32%; w/v) and 25 ml of NaOCl (50% available chlorine) were added to the hydrated cyst suspension to facilitate decapsulation. The process was stopped after 2 min by adding 35 ml of Na_2_S_2_O_3_ (10 g L^−1^). Filtered (0.22 μm) aeration was provided during the reaction. The decapsulated cysts were washed with filtered (passed through 0.45 μm membrane filter) and autoclaved (moist heat at 121°C for 15 min) artificial seawater (containing 35 g/L^-1^ of instant ocean synthetic sea salt, Aquarium Systems, Sarrebourg, France). Afterwards, the cysts were re-suspended in 500 ml of filtered, autoclaved seawater and hatched for at least 28 h at 28°C with constant illumination (c. 2000 lux). Air was bubbled through the suspension by a sterile glass tube extending to the bottom of the hatching vessel to keep all the cysts in continuous motion. The axenity of cysts was verified by inoculating 1 ml of culture water into 9 ml of TSB30 and incubating at 28°C for 24 h. After 28 h of hatching, batches of 30 larvae were counted and transferred to fresh, sterile 50 ml glass tubes containing 30 ml of filtered and autoclaved seawater. All manipulations were performed in a laminar flow to maintain sterility of the cysts and larvae. Finally, the tubes were incubated on a rotator (4 min^−1^) under 28°C.

The effects of the bacterial catecholamine antagonist on the virulence of *Vp*_AHPND_ strains were determined in a standardized challenge test with gnotobiotic brine shrimp larvae. The challenge tests were performed as described by Yang et al. (2014) with some modifications. *Vp*_AHPND_ was incubated overnight and cultures were washed with phosphate-buffered saline (pH 7.4) prior to inoculation into the brine shrimp rearing water at 10^6^ CFU ml^−1^, the bacterial catecholamine antagonist was added directly into the rearing water. Brine shrimp cultures without challenging with *Vp*_AHPND_ strains were used as controls. The survival of the larvae was counted 48 h after the addition of the pathogens. Each treatment was carried out in triplicate and each experiment was repeated three times to verify the reproducibility. In each test, the sterility of the control treatments was checked at the end of the challenge by inoculating 1 ml of rearing water to 9 ml of TSB30 and incubating the mixture for 2 days at 28°C.

### Conventional White Shrimp Challenge Test

Before the challenge test, healthy white shrimp (*Penaeus vannamei*; 4.6 ± 0.48 g) were transferred to 50 L seawater in plastic tanks, and were acclimated for one week under the following conditions: 30 shrimp/tank, natural photoperiod, seawater at salinity 30, pH 7.8, constant aeration and controlled temperature at 28 ± 1°C. Shrimp were fed with a commercial shrimp diet and water was exchanged at a rate of 30% per day to remove feces and food that was not ingested.

The immersion challenge tests were conducted according to Tran et al. (2013) with some modifications. Different *Vp*_AHPND_ strains were cultured in TSB30 at 28°C under constant agitation (100 min^−1^) with or without different catecholamines and corresponding antagonists until the OD_600_ reached 0.8, afterwards, the bacterial suspension was adjusted to 1×10^8^ CFU ml^−1^. The immersion procedure was carried out by immersing 20 shrimp in 6 L of this suspension for 15 min with aeration. Shrimp in the negative control group were immersed in sterile TSB30 broth. Following the immersion, the shrimp and bacterial suspension were transferred directly into an experimental tank containing 20 L of clean seawater to obtain an approximate bacterial density of 1×10^6^ CFU ml^−1^. The cumulative mortality was recorded every 2 h within 48 h.

### Activity Measurement of Immune-Related Enzymes

The activities of superoxide dismutase (SOD) and catalase (CAT) in the shrimp tissues from the bacteria challenge experiment were measured by the commercial kit (Nanjing, Jiancheng, A001-1 and A007) according to the operation instruction. Total SOD activity was determined by the hydroxylamine method. One SOD activity unit was defined as the enzyme amount causing 50% inhibition in 1 ml reaction solution. Total CAT activity was determined by using the spectrophotometry to measure yellowish complex compound yielded from the reaction between hydrogen peroxide and ammonium molybdate. One CAT enzyme activity unit was defined as the amount of protein to degradate 1 mmol hydrogen peroxide per second. The concentration of total protein in the supernate was quantified by BCA method. The SOD and CAT activities in supernate sample were the ratio of total enzyme activity unit to the total protein respectively, and the results were expressed as U mg^−1^ protein.

### Statistical Analysis

All data were presented as the mean ± standard deviation (SD). Data analysis was carried out using the SPSS statistical software (version 26); t-test or one-way analysis of variance (ANOVA) followed by Tukey’s *post hoc* tests was performed for between-group analyses. The survival of white shrimp was analyzed and expressed as a Kaplan–Meier survival curve. A probability (*p*) value <0.05 was considered as statistically significant, and a probability (*p*) value <0.01 was considered as extremely significant.

## Results

### QseC Mediates Catecholamine-Induced Effects on Growth and Motility in *Vp*_AHPND_

The bacterial receptor QseC, which is a membrane-bound histidine sensor kinase, has been reported to sense the bacterial signal autoinducer-3 (AI-3) and the host stress catecholamines epinephrine and/or norepinephrine (Clarke et al., 2006). Various bacterial pathogens have exploited the QseC signaling cascade to mediate virulence, such as enterohemorrhagic *E. coli* and *S. enterica* serovar Typhimurium (Rasko et al., 2008; Curtis et al., 2014; Moreira et al., 2016). Therefore, to verify the role of QseC in the pathogen response to catecholamines, we generated *qseC* in-frame deletion mutant (Δ*qseC*) and a complemented strain (*qseC+*).

**Figure 1:**
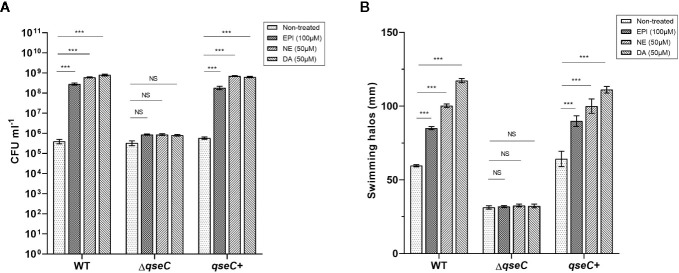
QseC mediates catecholamine-induced growth and flagellar motility in *Vp*_AHPND_ strains. **(A)** Viable counts of *Vp*_AHPND_ wild type 123, Δ*qseC* mutant and complementary strain *qseC*+ after 48 h of incubation from an inoculum of 10^2^ CFU ml^-1^ in serum-SAPI medium with catecholamines; **(B)** Sizes of motility halos after 18 h of incubation on TSA30 plates containing 0.3% agar with different catecholamines. Epinephrine (EPI), norepinephrine (NE) and dopamine (DA) were used at 100, 50, and 50 µM, respectively. Error bars represent the standard deviation of three independent cultures. Asterisks indicate significant differences when compared to untreated *Vp*_AHPND_ (independent samples t- test; *p < 0.05; **p < 0.01; ***p < 0.001).

The growth response of *Vp*_AHPND_ strains to different catecholamines was investigated in a minimal salts medium supplemented with 30% (v/v) adult bovine serum (serum-SAPI), which exposed *Vp*_AHPND_ to conditions similar to those inside a host, including limited nutrient availability, iron limitation, and immune defense antibodies. The results revealed that the addition of all three kinds of catecholamines tested (i.e., EPI, NE, and DA) could significantly increase the growth of *Vp*_AHPND_ 123 wild type and complemented strain, but not of Δ*qseC* mutant (**Figure 1A**). However, the catecholamines had no effect on the growth of *Vp*_AHPND_ strains in medium without serum (data not shown).

Flagellar motility is considered essential to enhance the initial interaction of a bacterium with a surface by enabling the cell to overcome negative electrostatic forces (Josenhans and Suerbaum, 2002). Several pathogenic species have been convincingly shown to require motility to infect a host, such as *V. parahaemolyticus*, *V. harveyi*, and *V. cholerae* (Krukonis and DiRita, 2003; Zhang and Orth, 2013; Yang and Defoirdt, 2015). Thus, we investigated the effect of QseC on catecholamines-induced swimming motility of *Vp*_AHPND_. All three catecholamines enhanced the flagellar motility of the wild type and complemented strain, while no stimulatory effect was observed in Δ*qseC* mutant (**Figure 1B**). These observations confirmed that QseC mediates the effects of catecholamines on *Vp*_AHPND_ growth and motility.

### Global Transcriptional Profile of Δ*qseC* Mutant

**Figure 2:**
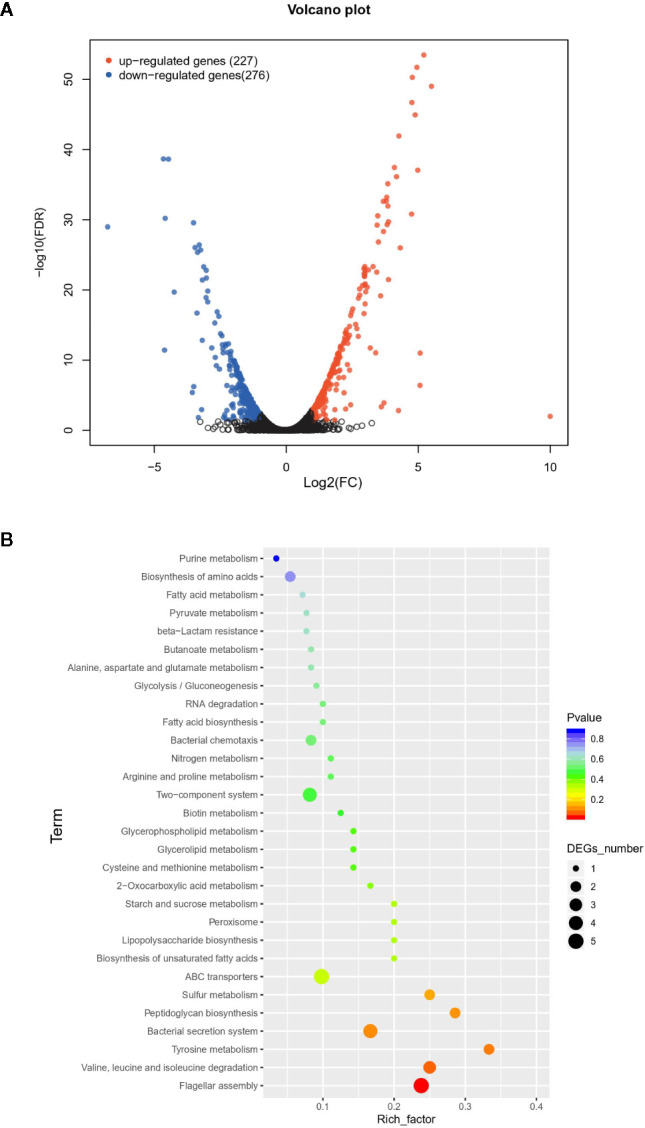
The difference between wild type and mutant strain at the whole transcriptome level. **(A)** Volcano plot of differentially expressed genes (DEGs). In total, 503 unigenes present different expression levels. Red indicates upregulated expression, green indicates downregulated expression and blue indicates no significant differential expression when comparing. **(B)** Functional enrichment of differentially expressed genes on KEGG categorization in mutant strain.

The difference between *Vp*_AHPND_ 123 wild type and Δ*qseC* mutant strain was also analyzed at the whole transcriptome level. In total, 503 unigenes displayed different expression levels, of which 227 were up-regulated and 276 were down-regulated (**Figure 2A**). To analyze and characterize the specific differentially expressed genes (DEGs), a false discovery rate less than 5% and fold change >1.5 was selected as limitation for significant difference. Results revealed that there were two groups of genes with distinct expression profiles. Group 1 (136 genes) was up-regulated in the mutant strain, 51.4% of which distributed in nine gene clusters (**Table 3**). Notably, 22 genes belonging to T6SS (Cluster 1) were up-regulated in the mutant strain, suggesting the deletion of *qseC* might result in a higher capacity to outcompete with other bacterial species. Another example of “unexpected” up-regulated response concerned the genes related to cell division (Cluster 4). The remaining seven clusters contained numerous genes controlling various cellular functions, three of which (clusters 1, 2, and 3) included a number of genes (see **Table S1**) primarily related to chromosome partition, while one cluster was involved with Copper-sensing two-component system (clusters 8), and four clusters associated with transport (clusters 5, 6, 7, and 9) were also over-expressed. In addition, a large number of genes in mutant strain involved in lipid metabolism were also up-regulated but without significant difference relative to wild type (fold change between 1.0 and 1.5) (**Table S1**).

**Table 3:** Genes up-regulated (> 1.5-fold change) in the mutant strain.

Cluster	Gene	logFC	P value	Function
1	VP1448	1.8407	8.78E-05	Anaerobic dimethyl sulfoxide reductase chain B
	VP1449	2.346482	1.05E-15	Anaerobic dimethyl sulfoxide reductase chain A
	VP1450	3.442818	2.86E-32	hypothetical protein
	VP1451	1.867141	1.08E-11	hypothetical protein
	VP1452	2.885653	2.51E-23	Outer membrane protein ImpK/VasF
	VP1453	2.951883	4.14E-25	Uncharacterized protein ImpJ/VasE
	VP1454	2.990298	1.34E-23	Type VI secretion lipoprotein/VasD
	VP1455	2.964036	9.35E-25	Uncharacterized protein ImpI/VasC
	VP1456	2.786061	6.17E-22	hypothetical protein
	VP1457	3.429694	2.33E-25	Uncharacterized protein ImpA
	VP1458	4.096527	7.38E-41	IcmF-related protein
	VP1459	2.94613	3.15E-19	Transcriptional regulator
	VP1460	1.667593	9.05E-10	Uncharacterized protein ImpH/VasB
	VP1461	2.279961	2.73E-16	Protein ImpG/VasA
	VP1462	2.129158	6.08E-14	Uncharacterized protein
	VP1463	2.518079	6.29E-20	Uncharacterized protein ImpC
	VP1464	2.393249	4.34E-16	Uncharacterized protein ImpB
	VP1465	2.476941	1.97E-19	Uncharacterized protein ImpA
	VP1466	2.440396	6.30E-19	Serine/threonine protein kinase
	VP1467	1.832535	6.93E-12	Hcp
	VP1468	2.980778	3.95E-25	ClpB-like protein
	VP1469	2.628548	1.12E-17	sigma-54-dependent transcriptional regulator
	VP1470	1.536542	0.003818	Uncharacterized peptidase YqjE
				
2	VP1740	2.30968	1.75E-15	FIG01199787: hypothetical protein
	VP1741	2.394921	2.28E-17	Chromosome segregation ATPase
	VP1742	2.438215	1.55E-05	hypothetical protein
				
3	VP1043	1.70837	3.81E-10	hypothetical protein
	VP1044	1.889812	7.64E-08	hypothetical protein
	VP1045	1.791027	1.62E-11	Chromosome partition protein MukB
	VP1046	1.945746	3.68E-12	Chromosome partition protein MukE
	VP1047	2.002129	2.34E-13	Chromosome partition protein MukF
	VP1048	2.21627	1.06E-15	S-adenosylmethionine-dependent methyltransferase
	VP1049	1.637211	5.65E-10	Positive regulator of competence TfoX
				
4	VP0461	1.533797	5.29E-09	UDP-N-acetylmuramate–L-alanine ligase
	VP0462	1.756452	9.66E-11	UDP-N-acetylglucosamine–N-acetylmuramyl
	VP0463	1.780125	2.75E-11	Cell division protein FtsW
	VP0464	1.740744	8.28E-11	UDP-N-acetylmuramoyl
	VP0465	1.743484	6.24E-11	UDP-N-acetylmuramoylalanyl-D-glutamate–2
	VP0466	1.889101	2.15E-12	Cell division protein FtsI
	VP0467	2.073686	1.80E-14	Penicillin-binding protein activator
	VP0468	1.984658	8.80E-13	Predicted endonuclease
				
5	VP0091	3.804372	1.85E-36	UPF0118 membrane protein SMc00793
	VP0092	3.821178	2.33E-32	FIG01201038: hypothetical protein
	VP0093	3.492542	8.14E-30	putative membrane protein
	VP0094	3.575819	8.18E-22	FIG01201571: hypothetical protein
	VP0095	4.757663	2.03E-50	DUF2383 domain-containing protein
	VP0096	2.97958	2.04E-23	FIG01200706: hypothetical protein
	VP0097	3.848639	1.97E-38	Methylglyoxal synthase
	VP0098	3.289054	3.31E-26	Hemerythrin domain protein
	VP0099	4.747694	5.81E-34	hypothetical protein
	VP0100	4.948999	8.06E-56	FIG01201373: hypothetical protein
	VP0101	4.774471	3.22E-54	inducible periplasmic protein
	VP0102	2.187439	4.88E-15	sigma-54 interacting response regulator
				
6	VPA0220	3.603139	3.24E-05	Protein ytfJ precursor
	VPA0221	3.013183	2.07E-22	Carbonic anhydrase alpha class
	VPA0222	3.386627	1.93E-13	Long-chain fatty acid transport protein
	VPA0223	4.883209	1.43E-48	Transcriptional regulator
	VPA0224	2.249278	6.81E-05	hypothetical protein
	VPA0225	2.317688	1.16E-11	Hemolysin
				
7	VP2662	1.671946	3.19E-10	Phospholipid ABC transporter
	VP2663	1.973801	2.06E-13	Phospholipid ABC transporter MlaC
	VP2664	1.781821	4.77E-11	Phospholipid ABC transporter MlaB
				
8	VPA0918	1.893487	1.55E-12	FIG01199656: hypothetical protein
	VPA0919	1.953746	2.73E-13	Copper-sensing two-component system response
	VPA0920	2.052521	2.29E-14	Signal transduction histidine kinase
				
9	Unknown	1.629797	1.10E-08	hypothetical protein
	Unknown	1.715916	2.12E-10	Outer membrane protein
	Unknown	1.505204	1.19E-08	hypothetical protein
	Unknown	1.50863	7.42E-09	hypothetical protein
	Unknown	1.942236	4.04E-13	hypothetical protein

In contrast, 135 genes in group 2 were down-regulated, 47.4% of which belonged to eight gene clusters (**Table 4**), including six genes encoding Sigma-fimbriae usher proteins, three genes encoding Arginine ABC transporter, three genes encoding Cytochrome c551 peroxidase, 15 genes related to Type II/IV secretion system, seven genes associated with flagellin biosynthesis, 10 genes associated with Glycosyltransferase, 15 genes encoding flagellin proteins, and five genes with unknown function. Genes related to biofilm formation such as *sypN* (encoding Glycosyltransferase), and *cpsA* (Capsular polysaccharide synthesis) were also down-regulated. Besides, the expression of several genes encoding Methyl-accepting chemotaxis protein, DNA protection during starvation protein and transcriptional regulator were also decreased.

**Table 4:** Genes down-regulated (> 1.5-fold change) in the mutant strain.

Cluster	Gene	logFC	pvalue	function
1	VPA1504	-2.15582	1.68E-11	Sigma-fimbriae tip adhesin
	VPA1505	-1.83097	3.17E-10	Sigma-fimbriae usher protein
	VPA1506	-1.85957	5.85E-10	Sigma-fimbriae usher protein
	VPA1507	-1.80196	6.91E-10	Sigma-fimbriae usher protein
	VPA1508	-1.66659	2.43E-09	Sigma-fimbriae usher protein
	VPA1509	-1.76698	1.29E-09	Sigma-fimbriae chaperone protein
				
2	VPA0636	-1.55671	1.22E-07	Arginine ABC transporter
	VPA0637	-1.69805	1.73E-09	Arginine ABC transporter
	VPA0638	-1.5677	3.61E-08	Arginine ABC transporter
				
3	VPA1099	-1.95092	7.45E-08	Predicted redox protein
	VPA1100	-2.42168	1.19E-14	Sensor histidine kinase
	VPA1101	-2.11042	2.77E-12	Cytochrome c551 peroxidase
				
4	VP2423	-4.58784	2.59E-33	hypothetical protein
	VP2424	-4.46142	4.30E-42	Type IV prepilin peptidase TadV/CpaA
	VP2425	-4.65177	3.56E-42	Type IV prepilin peptidase TadV/CpaA
	VP2426	-3.03105	1.31E-25	Predicted ATPase with chaperone activity
	VP2427	-3.02567	1.85E-24	Flp pilus assembly protein RcpC/CpaB
	VP2428	-2.55322	7.99E-19	Type II/IV secretion system secretin RcpA
	VP2429	-1.75485	0.000238	hypothetical protein
	VP2430	-1.97074	2.91E-12	Von Willebrand factor type A domain protein
	VP2431	-1.86147	6.08E-11	Similar to TadZ/CpaE
	VP2432	-1.78401	5.98E-10	Similar to TadZ/CpaE
	VP2433	-1.76889	2.37E-10	Type II/IV secretion system ATPase TadZ/CpaE
	VP2434	-1.73857	5.29E-10	Type II/IV secretion system ATP hydrolase TadA/VirB11/CpaF
	VP2435	-1.80475	2.63E-10	Flp pilus assembly protein TadB
	VP2436	-1.74251	1.77E-09	Type II/IV secretion system protein TadC
	VP2437	-1.7222	9.38E-10	TPR repeat protein
				
5	VP2261	-3.03897	1.46E-21	Flagellin protein FlaF
	VP2262	-3.36217	2.86E-28	Flagellin protein FlaD
	VP2263	-3.51179	1.21E-32	Flagellin protein FlaA
	VP2264	-3.24318	1.29E-28	Flagellar protein FlaG
	VP2265	-3.29545	2.24E-29	Flagellar cap protein FliD
	VP2266	-3.3816	2.61E-19	Polar flagellar rod protein FlaI
	VP2267	-2.97178	1.54E-22	Flagellar biosynthesis protein FliS
				
6	VP1657	-1.99881	3.80E-12	Type III secretion host injection protein (YopB)
	VP1658	-2.12616	3.16E-13	Type III secretion negative regulator protein (YopD)
				
7	VP1476	-2.68838	9.64E-13	Anti-sigma regulatory factor SypA
	VP1477	-1.58128	1.64E-07	Outer membrane protein SypB
	VP1478	-1.53419	1.10E-06	Predicted protein SypD
	VP1479	-1.6465	8.60E-05	Signal transduction histidine kinase SypF
	VP1480	-1.75662	7.30E-08	Oligosaccharide translocase SypK
	VP1481	-1.72636	5.83E-07	Membrane protein SypL
	VP1482	-2.82179	3.63E-14	Glycosyltransferase SypN
	VP1483	-3.18243	2.64E-15	SypO
	VP1484	-1.66319	4.82E-06	Glycosyltransferase SypQ
	VP1485	-3.20651	8.60E-05	Sugar transferase SypR
	VPA1403	-2.2481	0.000113	Capsular polysaccharide synthesis enzyme CpsA
	VPA1406	-1.54633	9.98E-06	Capsular polysaccharide synthesis enzyme CpsD
				
8	VP0791	-2.52929	5.65E-11	Flagellin protein FlaE
	VP0792	-3.17949	3.71E-24	Flagellin protein FlaD
	VP0793	-2.61879	1.70E-19	Flagellin protein FlaC
	VP0794	-2.43402	5.55E-16	Flagellar hook-associated protein FlgL
	VP0795	-2.2913	1.66E-14	Flagellar hook-associated protein FlgK
	VP0796	-2.39126	1.93E-13	Flagellar protein FlgJ [peptidoglycan hydrolase]
	VP0797	-2.70585	7.15E-18	Flagellar P-ring protein FlgI
	VP0798	-2.1387	7.01E-11	Flagellar L-ring protein FlgH
	VP0799	-2.49074	2.69E-16	Flagellar basal-body rod protein FlgG
	VP0800	-2.39727	4.95E-14	Flagellar basal-body rod protein FlgF
	VP0801	-1.86615	2.83E-11	Flagellar hook protein FlgE
	VP0802	-1.86572	3.50E-11	Flagellar basal-body rod protein FlgD
	VP0803	-1.88728	2.29E-11	Flagellar basal-body rod protein FlgC
	VP0804	-1.92001	8.89E-12	Flagellar basal-body rod protein FlgB
	VP0805	-3.55641	1.93E-07	Flagellar protein FlgP
	VP0806	-2.28843	9.85E-15	Flagellar protein FlgO

Overall, group 1 and group 2 comprised the majority of DEGs. The Kyoto Encyclopedia of Genes and Genomes (KEGG) enrichment analysis revealed that a large number of DEGs primarily related to ABC transporter, flagellar assembly, secretion systems, and two-component system (**Figure 2B**).

Since strain 123 harbored one *pirAB^VP^*-negative and one *pirAB^VP^*-positive plasmid, we further examined the effect of *qseC* mutation on the expression of genes in these two plasmids. For *pirAB^VP^*-negative plasmid, 12 genes were differentially expressed. However, none of them had significant difference (fold change < 1.5) except for ORF2 (encoding hypothetical protein), ORF3 (encoding outer membrane protein) and ORF9 (encoding hypothetical protein), indicating the deletion of *qseC* did not have significant impacts on the expression of genes in *pirAB^VP^*-negative plasmid (**Table S2**). Likewise, no genes including *pirAB^VP^* was up- or down-regulated significantly on *pirAB^VP^*-positive plasmid in Δ*qseC* mutant.

### QseC Is Involved in Bacterial Motility, Biofilm Formation, and Pathogenesis of *Vp*_AHPND_

**Figure 3:**
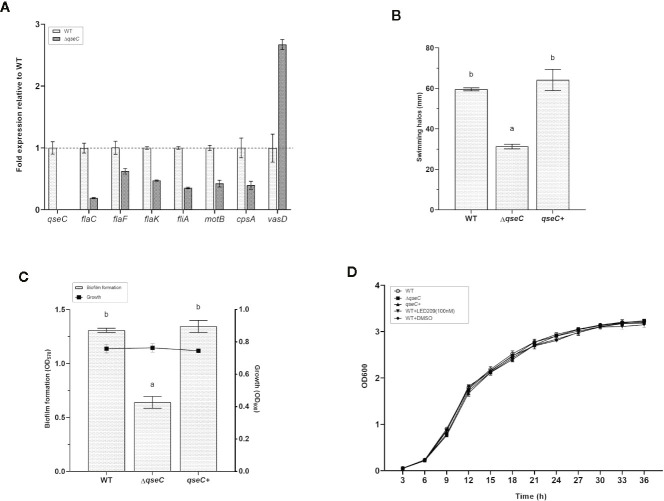
QseC mediates virulence-related gene expression, flagellar motility, biofilm formation, and T6SS in *Vp*_AHPND_. **(A)** RT-qPCR detection of expression of the virulence-related genes in wild type (WT) and Δ*qseC* mutant cultured to exponential phase. Genes normalized to *recA*, and fold change relative to wild type strain. All values were expressed as mean ± standard deviation (SD) of three biological replicates. **(B)** Swimming motility assays performed on soft agar plates after 21 h of incubation. The data were replotted from **Figure 1B**. Error bars indicated the SD of three independent experiments. Different letters indicated significant differences (one way ANOVA with Tukey’s *post hoc* test; *p* < 0.01). **(C)** Biofilm formation in different *Vp*_AHPND_ strains. Error bars indicated the SD of three independent experiments. Different letters indicated significant differences (one way ANOVA with Tukey’s *post hoc* test; *p* < 0.01). **(D)** Impact of *qseC* deletion and catecholamine receptor antagonist LED209 on the growth of *Vp*_AHPND_ 123. Error bars represented SD of three independent cultures.

Based on the transcriptomic analysis, several genes associated with flagellar motility, biofilm formation, chemotaxis, and T6SS were found to be differentially expressed in Δ*qseC* mutant. To confirm whether QseC regulates these virulence factors of *Vp*_AHPND_ 123, we further validate the results by RT-qPCR and also by a range of phenotypic screens. The expression of eight genes were verified in the wild type and mutant strains, including *qseC*, *cpsA* (relating to biofilm formation), *flaC* and *flaF* (encoding polar flagellins), *flaK* (encoding σ^54^-interacting regulator), *fliA* (encoding polar flagellin specific chaperon), *motB* (encoding Na^+^ motor component), and and *vasD* (associating with T6SS). Consistent with the transcriptomic results, all these genes were down-regulated in Δ*qseC* mutant (*p* < 0.01) except for *vasD*, which was up-regulated to 2.7-fold expression (**Figure 3A**). In addition, QseC expression was not detected in Δ*qseC*.

In the following experiments, we used soft agar plates and crystal violet staining to investigate the phenotypic differences in *Vp*_AHPND_ strains and to corroborate our transcriptional expression data. According to the results, the Δ*qseC* mutant exhibited motility defects based on a significantly smaller swimming halo compared with the wile type strain, while the deficiencies were restored in the *qseC+* complemented strain (**Figure 3B**). Similar results were observed in the biofilm formation assay, there was marked reduction of biofilm formation in Δ*qseC* mutant compared with the parental and complemented strains (**Figure 3C**), and substantiating the idea that QseC is involved in the regulation of *Vp*_AHPND_ flagellar motility and biofilm formation. Additionally, the presence and absence of *qseC* gene showed no effect on the growth of *Vp*_AHPND_ (**Figure 3D**).

Overall, QseC has been shown to regulate several virulence factors in *Vp*_AHPND_. However, the role it plays in pathogenesis *in vivo* has not been addressed. Given the fact that flagellar motility and biofilm formation are required for virulence of *V. parahaemolyticus*, we further assessed the virulence of Δ*qseC* mutant using a standard brine shrimp challenge test. According to the result, Δ*qseC* mutant is attenuated for virulence towards brine shrimp larvae (**Table 5**), thereby establishing that QseC is involved in pathogenesis of *Vp*_AHPND_.

**Table 5:** The impacts of *qseC* deletion and LED209 on the virulence of *Vp*_AHPND_ towards gnotobiotic brine shrimp larvae (*Artemia franciscana*).

Strains	Survival (%)^1^	
Control	100 ± 0	
		
*Vp*_AHPND_ WT	32 ± 8	a
*Vp*_AHPND_ Δ*qseC*	57 ± 3	c
		
*Vp*_AHPND_ [5 pM LED209]	38 ± 3	a
*Vp*_AHPND_ [5 nM LED209]	47 ± 8	b
*Vp*_AHPND_ [50 nM LED209]	60 ± 5	c

^1^Survival rate of brine shrimp larvae after challenging with Vp_AHPND_ strain for 48 h.

Survival in the negative control was set at 100% and the other treatments were normalized accordingly. Batches of 30 brine shrimp larvae were used for each replicate, and each treatment was carried out in triplicate. Square brackets refer to pretreatment. LED209 was directly added into the Artemia rearing water. Data are average ± standard deviation of three independent experiments, and were analyzed by One-way ANOVA with Tukey’s post hoc test (p < 0.01). Values with a different letter are significantly different from each other.

### LED 209 Inhibition of QseC as a Promising Antivirulence Strategy

**Figure 4:**
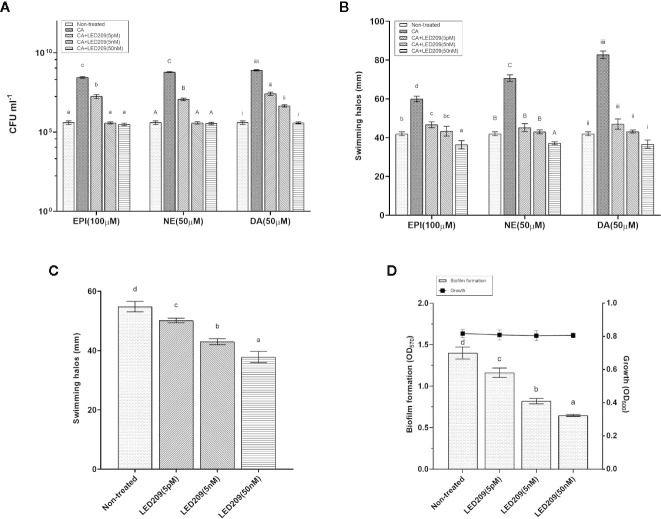
LED209 neutralizes the catecholamine enhanced growth **(A)** and swimming motility **(B)** in *Vp*_AHPND_ 123. Three kinds of catecholamine including Epinephrine (EPI), norepinephrine (NE), and dopamine (DA) were used at 100, 50, and 50 µM, respectively. **(C)** Treatment with LED209 diminished swimming motility of *Vp*_AHPND_ 123. Diameters of motility halos after 18 h of incubation on TSA30 plates containing 0.3% agar were measured in the presence (5 pM, 5 nM, and 50 nM, respectively) or absence (DMSO only) of LED209. **(D)** Administration of LED209 reduced biofilm formation of *Vp*_AHPND_ 123. Strain was grown statically in TSB30 in 96-well plate for 24 h in the presence (5 pM, 5 nM, and 50 nM, respectively) or absence (DMSO only) of LED209. Biofilm formation was determined by measuring crystal violet binding at OD_570_. The error bars represented standard deviation (SD) of three independent experiments. Different letters indicate significant differences (One-way ANOVA with Tukey’s *post hoc* test; *p* < 0.01).

LED209 is a specific antagonist that can inhibit the binding of signaling molecules to QseC, thus prevents its autophosphorylation and consequently blocks QseC-mediated virulence gene expression (Rasko et al., 2008). According to our results, LED209 was able to neutralize the catecholamine-induced effects of EPI, NE, and DA on *Vp*_AHPND_ 123 growth and motility (**Figures 4A, B**), which confirms its role on QseC inhibition. Besides, the addition of LED209 could also significantly decrease swimming motility and biofilm formation in *Vp*_AHPND_ 123 without exogenous catecholamines (**Figures 4C, D**), this result echoes our previous observation showing that Δ*qseC* mutant exhibited motility and biofilm defects. Most importantly, our results revealed that LED209 reduced the virulence of *Vp*_AHPND_ 123 towards brine shrimp larvae (**Table 5**), while did not influence its growth *in vitro* (**Figure 3D**).

**Figure 5:**
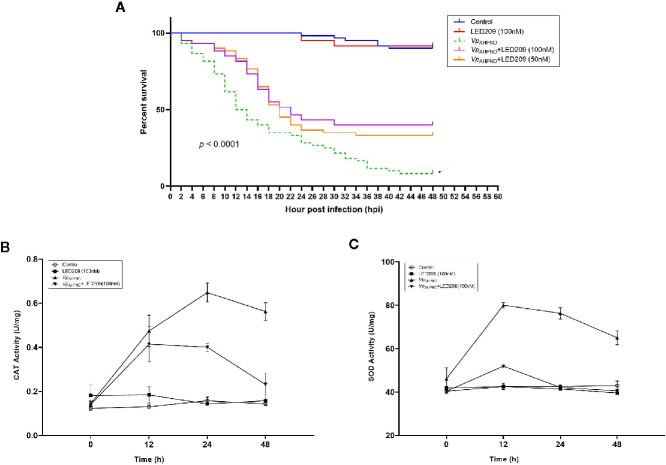
Dynamics of mortalities and temporal activities of the immune-related enzymes after *Vp*_AHPND_ challenge. **(A)** Treatment of LED209 protects white shrimp (*Penaeus vannamei*) from *Vp*_AHPND_ infection. LED209 was used at 50 and 100 nM, respectively. The survival curves were recorded for 48 h after challenge, *p* < 0.0001 based on Kaplan-Meier survival analysis with a log rank test with Prism software (Graphpad Software). **(B)** Superoxide dismutase (SOD) activity. **(C)** Catalase (CAT) activity. Data were presented as mean ± standard deviation (SD) of three independent experiments. (*Note: the survival curve data of *Vp*_AHPND_ 123 without treatment have been published in another article by the author (Yang et al., 2020, (J) Appl. Microbiol.), and the relevant data were obtained from the same batch of experiments.)

Based on these promising results, we went further to investigate the effect of LED209 on the survival of challenged white shrimp (*P. vannamei*), a commercially important aquaculture species. According to our previous research, only 8% of shrimp challenged with untreated V*p*_AHPND_ 123 remained alive at 48 hpi (Yang et al., 2010), while in this study, 40 and 47% of shrimp were still alive after challenging by the *Vp*_AHPND_ treated with 50 and 100 nM LED209, respectively (**Figure 5A**). Moreover, LED209 showed no evidence of toxicity towards the shrimp, revealed by the survival rate of the group treated with LED209 reached 92% after 48 hpi.

Furthermore, mortality was delayed in LED209 treatment groups after infection with *Vp*_AHPND_ in comparison with the onset of the disease in the group without LED209. Mortality in the groups treated with LED209 began to increase at 10 hpi, and reached 56 and 52% in the group treated with 50 and 100 nM of LED209 during the following 24 h, respectively. After 30 hpi, no additional mortalities were observed. In contrast, for the group without LED209, mortality began to increase at 4 hpi and continued for an additional 40 h until reached a maximum of 92%. These observations demonstrated that LED209 can attenuate bacterial virulence without interfering with the growth, which makes it a potential antivirulence agent.

### The Activity Change of Immune-Related Enzymes in the Shrimp After Bacterial Challenge

We also monitored the activity change of immune-related enzymes including SOD and CAT in the shrimp after challenge. The activities of CAT in the challenged group increased significantly during 24 h, and reached the peak (0.677 U mg^-1^, *p* < 0.05) at 24 h after challenge (**Figure 5B**). For the groups treated with LED209 (100 nM), it began to increase (0.401 U mg^-1^, *p* < 0.05) at 12 h and maintained the peak at 24 h after challenge. Similar observation was also identified for SOD activity (**Figure 5C**). The activities of SOD in the challenged group reached the peak (84 U mg^-1^, *p* < 0.05) at 12 h and were significantly higher than that in the treatment group with LED209 (100 nM) after challenge. There was no significant difference in the activities of SOD and CAT between the control group and the group treated with LED209 (100 nM) during the whole experiment.

## Discussion

It is widely acknowledged that the extensive use of antibiotics in shrimp farming practices contributes to the development of multidrug-resistant bacteria, which has constituted one of the greatest challenges in combating bacterial infection, and posed high risks to the environment and human health (WHO, 2018). Therefore, antivirulence therapy - the use of antimicrobial agents that specifically target the regulation pathways required for the pathogenesis, but not for microbial viability—has received particular attention. QseC is a conserved histidine sensor kinase of the QseBC two-component system presented in at least 25 important pathogens, and it has been demonstrated to activate the expression of virulence genes by sensing and responding to the host catecholamine hormones (Rasko et al., 2008). Given these facts, further investigation of QseC in the pathogenesis of *Vp*_AHPND_ may be important for developing novel strategies to combat AHPND.

In this study, we found that the wild type strain *Vp*_AHPND_ 123 but not Δ*qseC*, exhibited catecholamine enhanced growth and flagellar motility, indicating that QseC functions as a bacterial catecholamine receptor in *Vp*_AHPND_. These results manifest that QseC might play a role in the regulation of host adaptation processes of *Vp*_AHPND_ by utilizing the catecholamines or other hormones.

Primary metabolism is one of the essential phenotypes that significantly contribute to the virulence, depending on the availability of food sources and environmental dynamics (Peyraud et al., 2018). Transcriptome analysis revealed that QseC plays an essential role in the global gene regulation of *Vp*_AHPND_. The up-regulation of genes encoding lipid biosynthesis and transportation could imply that *Vp*_AHPND_ might try to deal with an excess of lipids accumulated due to the trade-off between virulence and primary metabolism (Peyraud et al., 2016). Besides, transcriptome analysis showed that T6SS gene cluster was strongly up-regulated. In addition to the alterations in expression of T6SS, another affected gene cluster associated with cell wall biosynthesis was identified. The underlying mechanism for the up-regulation of these genes remains unclear and is worth further attention. Taken together, these results suggest that QseC is a bacterial adrenergic receptor for *Vp*_AHPND_ that is crucial for environmental adaptation.

Transcriptome analysis also revealed the involvement of *qseC* in pathogenesis of *Vp*_AHPND_. Flagellum is an important surface organelle required for bacterial motility and host invasion. In the early stage of infection, flagellar motility promotes the translocation and colonization of pathogens within the host (Josenhans and Suerbaum, 2002). Elevated motility also helps the pathogen to more efficiently acquire nutrients and avoid toxic substances and facilitates dispersal in the environment during the course of transmission (Ottemann and Miller, 1997). In consistent with the findings in other pathogenic bacteria (Wang et al., 2011; Yang et al., 2014), our data indicated that expressions of various flagellar related genes were down-regulated in Δ*qseC* mutant. The investigation on soft agar plates confirmed that Δ*qseC* mutant displayed significantly impaired motilities and the deficiencies were rescued by complementation of the *qseC* gene.

Furthermore, similar results were observed in another assay indicating that Δ*qseC* showed weaker biofilm formation ability than the wild type and complementary strain. Transcriptome analysis also showed that genes related to capsular polysaccharide synthesis and glycosyltransferase were down-regulated in the mutant, suggesting the potential role of *qseC* in regulating biofilm formation. Bacterial biofilm formation is an important pathogenic mechanism that is involved in adherence, colonization, competence, metabolism, and stress response (Jefferson, 2004). Previous studies have demonstrated that QseC controls biofilm formation of different pathogens, such as *Haemophilus parasuis* and enteroaggregative *E.coli* (Curtis et al., 2014; He et al., 2018). At last, the observation that the virulence of Δ*qseC* mutant towards gnotobiotic brine shrimp larvae is attenuated, further underscores the role of QseC in *Vp*_AHPND_ pathogenesis.

Thus, our results verified the idea that QseC is a modulator of the host-pathogen interaction, and also an important regulator of *Vp*_AHPND_ virulence, all of which emphasize the need for further investigation into disturbing QseC as an approach to prevent *Vp*_AHPND_ infection. LED209 has been identified as an inhibitor that is highly specific for QseC by Rasko et al. (2008). It acts as a prodrug that can allosterically modify QseC, impeding virulence in several pathogens without interfere with the growth. More excitingly, it possesses desirable pharmacokinetics and does not exhibit toxicity *in vitro* and *in vivo* (Curtis et al., 2014). Therefore, we investigated whether inhibition of QseC with LED209 could reduce virulence of *Vp*_AHPND_ in two models, i.e., gnotobiotic brine shrimp larvae (*Artemia franciscana*) and conventional cultured white shrimp (*P. vannamei*). Intriguingly, we observed an attenuation of virulence by LED209 in both assays, even at a low concentration (nM). Unlike conventional antibiotics, LED209 does not kill or hinder *Vp*_AHPND_ growth, which makes it a promising antivirulence agent (Rasko et al., 2008).

In general, as bacterial histidine sensor kinases, the class of QseC signal transduction molecules are absent in mammals (Roychoudhury et al., 1993; Lyon and Muir, 2003), which makes it an attractive antivirulence therapy targets. QseC allows pathogens to sense and respond to catecholamine host hormones, and subsequently modulate their virulence gene expression for optimal host colonization. Meanwhile, it also helps the pathogens to detect the health state of the host, thereby invading or leaving (Curtis et al., 2014). Since these processes are not involved in bacterial growth, possibly leading to a lower evolutionary pressure for the drug resistance development. Furthermore, given the broad distribution of QseC in various Gram-negative pathogens and its inherent role in bacterial pathogenesis, LED209 may have promising application prospect as a broad-spectrum antivirulence agent.

A practical strategy is to combine LED209 with other available antibacterial drugs to design targeted dual-function antibacterial drugs. This novel dual-function antibacterial molecule can block QseC and reduce bacterial pathogenicity while targeting bacteria to improve treatment efficacy and reduce adverse reactions. Moreover, the likelihood of developing resistance to this virulence-regulating drug is relatively low. In the future, we will further investigate how LED209 reduces the pathogenicity of *Vp*_AHPND_ and clarify specific molecules and signal pathways in order to deepen our understanding of the mechanism of the QseC inhibition and provide a new strategy for treatment of AHPND.

## Conclusion

In conclusion, our study showed that QseC mediated the catecholamine stimulated effects on growth and flagellar motility of *Vp*_AHPND_. Transcriptome analysis of the wild type and Δ*qseC* mutant of *Vp*_AHPND_ confirmed the deletion of *qseC* suppressed flagellar motility, biofilm formation and virulence, but enhanced the expression of genes associated with T6SS and cell wall biosynthesis. The bacterial catecholamine receptor antagonist LED209 not only neutralized the effects of catecholamine but also attenuated the virulence of *Vp*_AHPND_ towards brine shrimp larvae and white shrimp, highlighting that interfering QseC with LED209 is a promising strategy to develop broad-spectrum antivirulence agents. These results provide insight into the use of an antivirulence approach for targeting not only *Vp*_AHPND_, but also a much larger collection of pathogenic bacteria.

## Data Availability Statement

The raw data supporting the conclusions of this article will be made available by the authors, without undue reservation.

## Ethics Statement

All applicable international, national, and/or institutional guidelines for the care and use of animals were followed.

## Author Contributions

JH and QY designed the study and coordinated the project. PZ, ZC, QW, and GX conducted the experiments. SF and QY conducted the transcriptome analysis. SF and QY wrote the manuscript. All authors contributed to the article and approved the submitted version.

## Funding

This work was supported by the National Natural Science Foundation of China (Grant No. 31702385), the Special Research Fund of Ghent University (BOF-UGent), the National Natural Science Foundation of China (Grant No. 81903372), and the China Agriculture Research System (CARS-48).

## Conflict of Interest

The authors declare that the research was conducted in the absence of any commercial or financial relationships that could be construed as a potential conflict of interest.
